# Associations between cerebral and systemic endothelial function in migrane patients

**DOI:** 10.1186/1129-2377-14-S1-P109

**Published:** 2013-02-21

**Authors:** JPO Pretnar-Oblak, BZ Zvan, MZ Zaletel

**Affiliations:** 1 University Clinical Center Ljubljana, Slovenia

## 

There is a growing interest in the role of endothelium in migraine patients. Recently, our group showed different endothelial function of the anterior and posterior cerebral circulation in healthy subjects and diminished posterior cerebral endothelial function in migraine patients without comorbidities. However, a relationship between cerebral and systemic endothelial function in migraine patients is still not clear. We compared cerebral and systemic endothelial function through post-hoc correlation analysis between cerebrovascular reactivity (CVR) to L-arginine in the anterior and posterior cerebral circulation, flow mediated vasodilatation (FMD) and intima-media thickness (IMT) in migraine patients without comorbidities. We did not find any significant correlation between CVR to L-arginine in the cerebral arteries (middle, posterior) and FMD in migraine patients with aura (p = 0.880 vs. p = 0.682), without aura (p = 0.153 vs. p = 0.179) and healthy subjects (p = 0.869 vs. p = 0.662). Also, IMT did not correlate with CVR to L-arginine in migraine patients with aura (p = 0.596), without aura (p = 0.414) and healthy subjects (p = 0.791). We found a significant correlation in CVR to L-arginine between the middle and posterior cerebral artery in migraine patients with aura (p = 0.004), without aura (p = 0.001) and healthy subjects (p = 0.002).

## Conclusion

Our study suggests that cerebral and systemic endothelial function is not associated in migraine patients, while endothelial. These results are valuable for better understanding the role of endothelial function in migraine patients.
